# Development of radioprotective side shield attached to radioprotective glasses for reducing eye lens dose of endoscopists during endoscopic retrograde cholangiopancreatography: a phantom study

**DOI:** 10.1007/s12194-025-00983-2

**Published:** 2025-11-03

**Authors:** Kotaro Fukushima, Kazuhiro Yamaguchi, Kosuke Matsubara

**Affiliations:** 1 Department of Quantum Medical Technology, Division of Health Sciences, Graduate School of Medical Sciences, Kanazawa University, 5-11-80 Kodatsuno, Kanazawa, 920-0942 Ishikawa Japan; 2 Kaz corporation, Co. Ltd, 3-23-24 Miyuki, Fukui, 910-0854 Fukui Japan; 3 Department of Quantum Medical Technology, Faculty of Health Sciences, Institute of Medical, Pharmaceutical and Health Sciences, Kanazawa University, 5-11-80 Kodatsuno, Kanazawa, 920-0942 Ishikawa Japan; 4 Present Address: Department of Radiological Technology, Toyota Kosei Hospital, 500-1 Ibobara Josui, Toyota, 470-0396 Aichi Japan

**Keywords:** Radiation protection, Endoscopic retrograde cholangiopancreatography, Occupational exposure, Eye lens, Radioprotective equipment

## Abstract

The use of radioprotective glasses alone may not reduce radiation sufficiently to protect the eyes of endoscopists performing endoscopic retrograde cholangiopancreatography (ERCP). In this study, we aimed to develop a radioprotective side shield that could be attached to radioprotective glasses. Air kerma was measured at the lens surface of an endoscopist phantom using optically stimulated luminescence dosimeters in an arrangement that simulated ERCP. The protective effect of the radioprotective side shield was evaluated by changing three factors: the number of sheets to be layered from 1 to 5, the front-side length of the sheet from 6 to 10 cm, and the rear-side length of the sheet from 5 to 7 cm. By increasing the front length of the sheets to 8 cm, their rear length to 6 cm, and increasing the number of sheets to three with a lead equivalent of 0.14 ± 0.02 mmPb, the air kerma on the ocular surface could be reduced considerably; hence, these changes were implemented. This achieved a dose reduction of 48.2–87.3% compared with the use of radioprotective glasses alone, especially when the endoscopist phantom was rotated 60° and 75° from the direction directly opposite to that of the patient phantom. The results suggest that radioprotective side shields can reduce radiation exposure in the eyes of endoscopists by < 0.03 mGy in 10-min fluoroscopy under most conditions during ERCP.

## Introduction

The eye lens is a highly radiosensitive tissue. Radiation cataracts resulting from performing X-ray fluoroscopy are widely reported by healthcare personnel [1]. The International Commission on Radiological Protection (ICRP) Publication 118 [2] set 0.5 Gy as the threshold dose for radiation cataracts, and has recommended that the new equivalent dose limit for the eye lens during occupational exposure be reduced from 150 mSv/y to an average of 20 mSv/y for five consecutive years, without exceeding 50 mSv in any one year. These guidelines support the need to protect the eye lenses of healthcare workers, maintaining exposure to radiation within the appropriate dose limits.

Endoscopic retrograde cholangiopancreatography (ERCP) is a fluoroscopy-guided procedure in which the bile and pancreatic ducts are visualized using a radiocontrast medium [3]. The necessity for ERCP is rising with an increase in common bile duct stones and biliary and pancreatic tract cancers in aging populations. In addition, the number of therapeutic ERCP procedures, such as endoscopic sphincterotomy (EST), has increased significantly and generally requires longer examination times than diagnostic ERCP [4–6]. Therefore, endoscopists performing these procedures tend to be exposed to higher radiation doses. Furthermore, over-couch fluoroscopy systems are commonly used during ERCP in many medical facilities. This system has the advantage of providing a larger working space above the patient than the under-couch system, making it easier to reposition the patient and apply manual abdominal compression during the examination. However, some studies have reported that the use of an over-couch fluoroscopy system results in a higher radiation dose to the endoscopist’s eye lens. Compared with the under-couch system, scattered X-rays generated by the patient mainly spread upward, increasing the amount of scattered X-rays reaching the direction of the endoscopist’s head [7–14]. Thus, unless endoscopists performing ERCP take the appropriate radiation protection measures, annual eye lens doses risk exceeding 20 mSv [10, 15].

Radioprotective glasses are a common method of lens protection used by healthcare workers. Radioprotective glasses are lightweight and designed to be comfortable even when worn for long periods, making them widely used not only in fluoroscopy-guided endoscopic procedures but also in vascular interventional radiology (IR). Several studies have examined the effect of radioprotective glasses on vascular IR, with the results showing that the use of radioprotective glasses can effectively reduce the lens dose [16–20]. However, in fluoroscopy-guided endoscopic procedures such as ERCP, radioprotective glasses may not be sufficient to prevent scattered X-rays generated by the patient from reaching the lens of the endoscopist’s eyes, as scattered X-rays enter through the gap between the endoscopist’s face and the radioprotective glasses. As endoscopists perform the procedure while looking more at the fluoroscopy monitor than at the patient, radioprotective glasses are unable to adequately shield against scattered X-rays [14, 21]. This positioning results in a larger angle of the endoscopist’s body relative to the patient, causing scattered X-rays to enter the eye from the side and diagonally behind the endoscopist. To overcome this disadvantage, a radioprotective side shield was developed that can be attached to any existing type of radioprotective glasses, which have already been adopted by facilities and healthcare workers. The radioprotective side shield is made of highly flexible olefin resin and incorporates deformable steel wires, allowing it to fit closely to the endoscopist’s face. In addition, it is lightweight (15.4 g) to ensure comfort during prolonged use.

In this study, we aimed to develop a radioprotective side shield that can be attached to radioprotective glasses and evaluate the dose-reduction effect of the eye lens in a phantom study simulating ERCP.

## Materials and methods

### Measurement of eye lens dose for endoscopist phantom

Using a patient phantom (Alderson Phantom Patient; Radiology Support Devices, Long Beach, CA, USA) and an island-type over-couch fluoroscopy X-ray system (AXIOM Luminos dRF; Siemens Healthineers, Erlangen, Germany), the air kerma at the left ocular surface of the endoscopist phantom was measured with small optically stimulated luminescence (OSL) dosimeters (nanoDot; Landauer, Glenwood, IL, USA) in an environment simulating ERCP. The small OSL dosimeters were read using a MicroStar reader (Landauer). The measurements were performed three times under the same conditions, and the small OSL dosimeters were read three times to reduce random errors [22]. Here, all air kerma measured with small OSL dosimeters were multiplied by a sensitivity correction factor of 0.75–1.00 (median 0.84), which was previously obtained for 73-kV X-rays with a 0.2-mm Cu additive filter using an ionization chamber (DC300; IBA Dosimetry, Schwarzenbruck, Germany) connected to an electrometer (EMF520R; EMF Japan, Hyogo, Japan). The ionization chamber was calibrated by the Japan Quality Assurance Organization using 70-kV X-rays (half-value layer 3.0 mm).

**Fig. 1:**
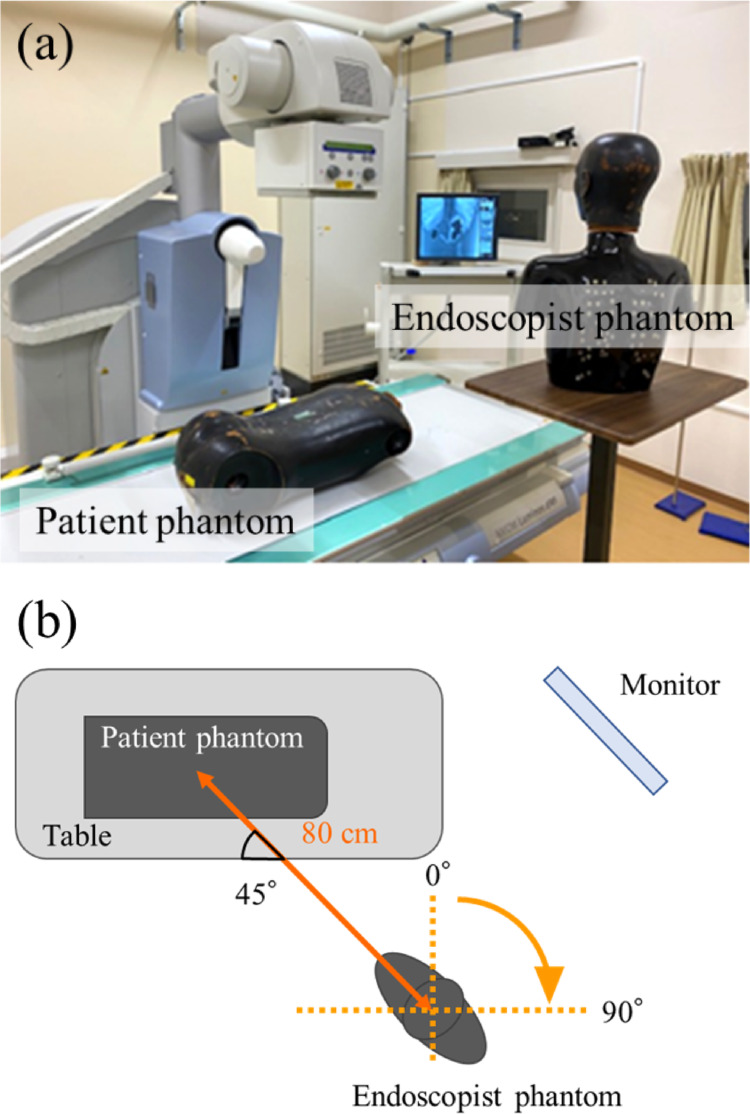
Arrangement assuming endoscopic retrograde cholangiopancreatography (ERCP), with the endoscopist phantom rotated clockwise to 45° from the position perpendicular to the long axis of the table (defined as 0°). **a** Photograph and **b** schematic of the experimental setup

The patient phantom was placed on the table, and the endoscopist phantom was placed 80-cm away from the center of the irradiation field and oriented 45° clockwise, perpendicular to the long axis of the patient phantom (Fig. 1). The table height was 80 cm, the distance from the X-ray tube to the image receptor was 115 cm, and the height of the endoscopist phantom varied between 150, 165, and 180 cm. Here, the body orientation of the endoscopist phantom was changed clockwise from 15° to 75° in 15° intervals, with the direction perpendicular to the long axis of the table set as 0°. This is because of the fact that endoscopists typically orient their bodies in different directions during the procedure, such as toward the patient and the fluoroscopy monitor screen. During the procedure, since the fluoroscopy monitor screen is often situated above the patient’s head, the endoscopist’s body orientation might result in this geometry [10, 15, 21]. The patient phantom was irradiated with X-rays for 10 min in pulsed fluoroscopy mode. The following fluoroscopic conditions were used, which were the system defaults for ERCP: tube voltage 73 kV (half-value layer 5.18 mm), tube current 26.9 mA, pulse width 8.1 ms, pulse rate 15 pps, additional filter 0.2 mm Cu, and irradiation field 29.8 × 29.8 cm.

### Determining the number of sheet layers and sheet size

#### Number of sheet layers and eye lens dose

The sheet was made of an olefin resin containing bismuth oxide (Bi_2_O_3_). The density of Bi_2_O_3_ was 2.20 g/cm^3^, and the thickness of each sheet was 0.35 mm. Air kerma was measured by varying the number of layers of the sheets from one to five. The length of the sheet was 8 cm on both the side closest to the face (hereafter referred to as the front side) and that closest to the occipital region (hereafter referred to as the rear side), and 0.85-mmPb radioprotective glasses (Dr. View X-RAY type OG; Kaz corporation, Fukui, Japan) were used. This type of glasses, which has smaller side lenses than the PANORAMA SHIELD HF-480S used in other sections, was chosen to focus on determining the shielding effect of the sheets without the additional protection provided by the glasses. The height of the endoscopist phantom was 165 cm, and the phantom was rotated from 45–75° at 15° intervals, with the direction perpendicular to the long axis of the table set as 0°, as described above. At 15° and 30°, the air kerma remained high regardless of the number of sheets, and these angles were therefore excluded, as this was considered to result from scattered X-rays entering through the gap below the front lens, since glasses had no lower lenses. The dose reduction rate (DR[%]) of the shield was calculated as follows:

1 $$\:\mathrm{D}\mathrm{R}\left[\%\right]=\frac{{K}_{\mathrm{G}}-{K}_{\mathrm{G}\mathrm{S}}}{{K}_{\mathrm{G}}}\times\:100$$

where $\:{K}_{\mathrm{G}}$ represents the air kerma when wearing only radioprotective glasses and $\:{K}_{\mathrm{G}\mathrm{S}}$ represents the air kerma when wearing radioprotective glasses with the shield.

#### Length of the front side of the sheet and eye lens dose

**Fig. 2:**
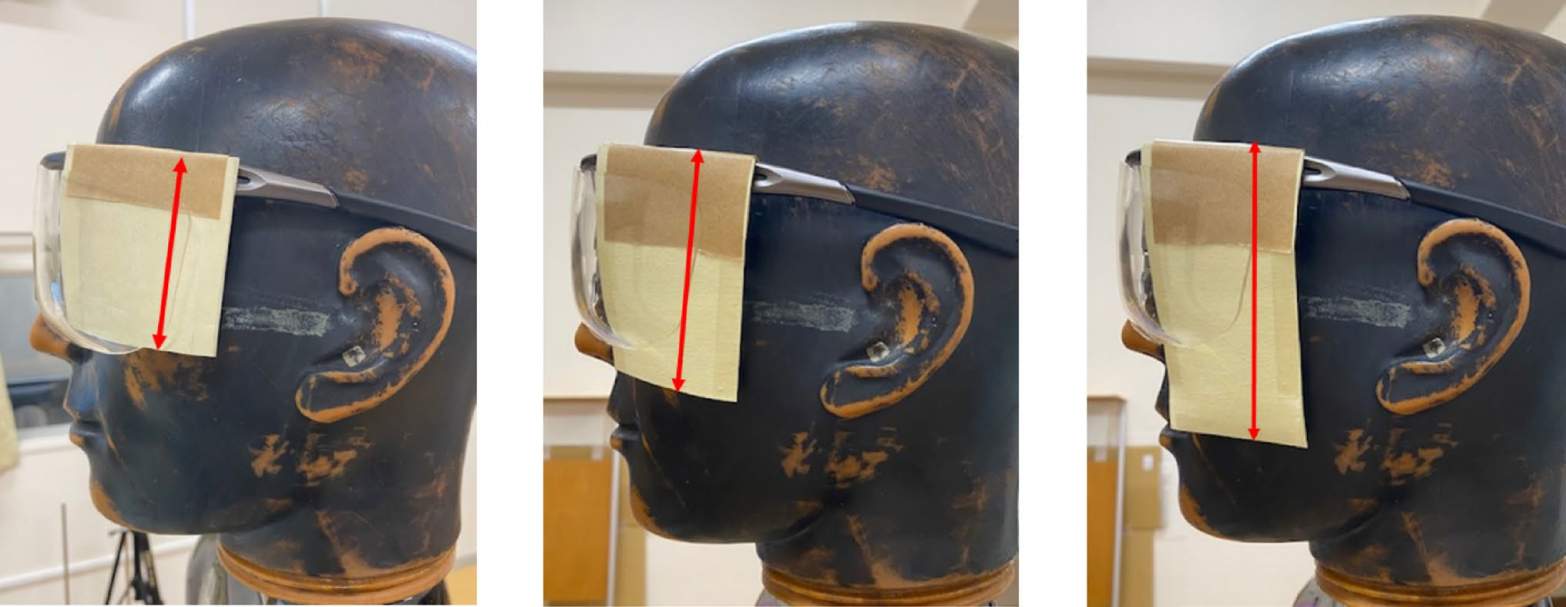
Radioprotective side shield of different lengths attached to radioprotective glasses (from left to right: 6, 8, and 10 cm)

The air kerma at the ocular surface of the endoscopist phantom was measured using sheets of 6 to 10 cm in length (Fig. 2), attached at 1-cm intervals. These sheets extended beyond the side lenses of the radioprotective glasses to provide additional shielding. The sheet was attached to the temple of 0.07-mmPb radioprotective glasses (PANORAMA SHIELD HF-480S; Toray Medical, Tokyo, Japan). The height of the endoscopist phantom was changed to 150, 165, and 180 cm, and rotated from 15–75° at 15° intervals. The DR[%] was calculated using Eq. (1).

#### Length of the rear side of the sheet and eye lens dose

**Fig. 3:**
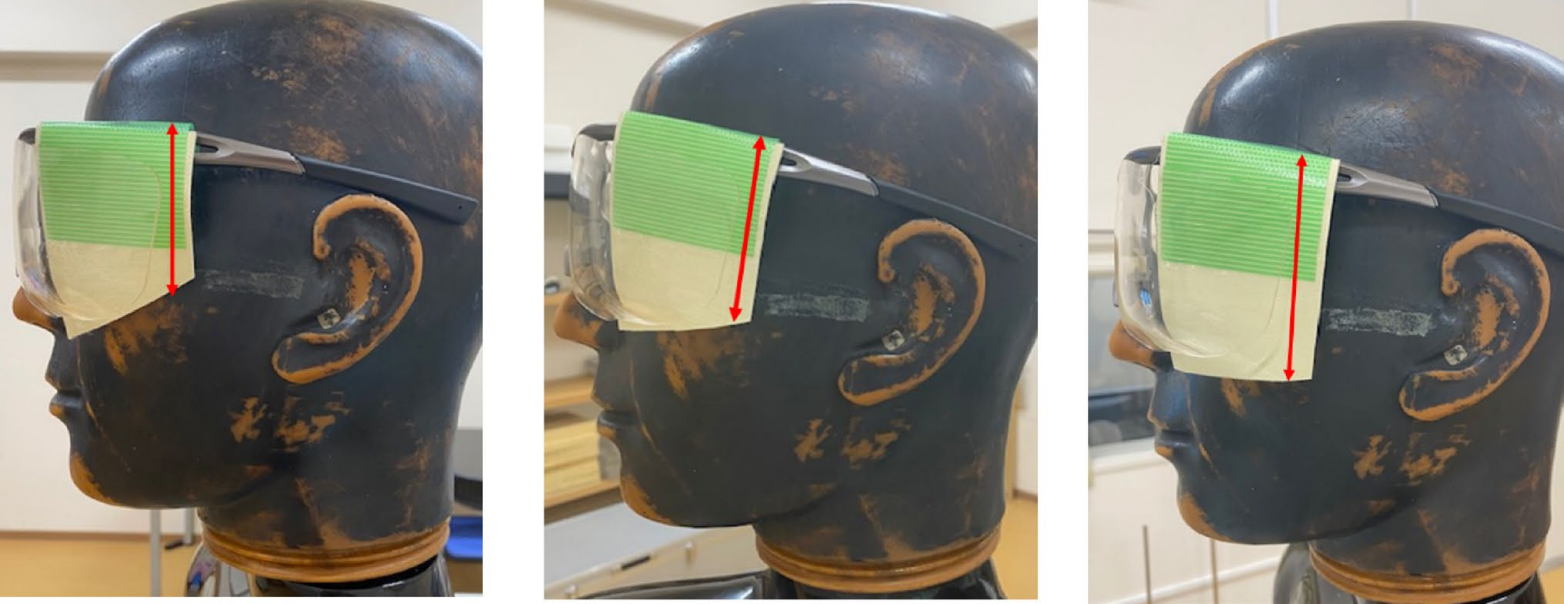
Radioprotective side shield of different rear lengths attached to radioprotective glasses (from left to right: 5, 6, and 7 cm)

The air kerma was measured by fixing the front-side length of the sheet at 8 cm and varying the rear-side length of the sheets from 5 to 7 cm at 1-cm intervals (Fig. 3). The same radioprotective glasses described in the previous section were used. The height of the endoscopist phantom was 165 cm or 180 cm, and the phantom was rotated from 15–75° at 15° intervals. The DR[%] was calculated using Eq. (1).

### Dose reduction effect of radioprotective side shield

**Fig. 4:**
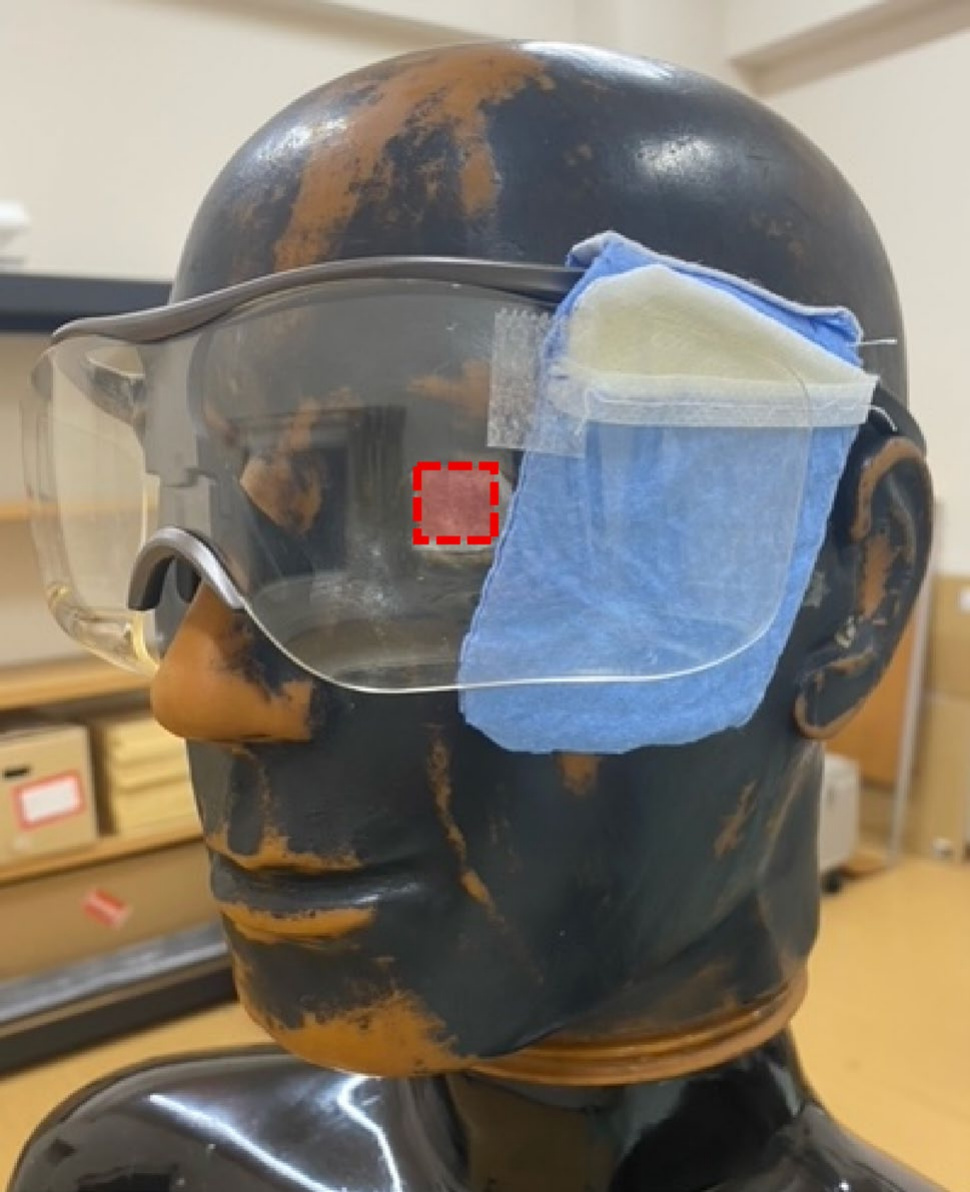
Photograph of radioprotective side shield attached to radioprotective glasses. The OSL dosimeters were attached to the left ocular surface of the endoscopist phantom at the position outlined with red dotted lines. The sheet was placed in a non-woven fabric case and attached to the radioprotective glasses by placing the case over the temple

The radioprotective side shield was composed of three layers of sheets with a front-side length of 8 cm and a rear-side length of 6 cm. The lead equivalent of the shield was 0.14 ± 0.02 mmPb for 120-kV narrow beam X-rays, in accordance with International Electrotechnical Commission (IEC) 61331-1 [23], with three stacked sheets, the layers being placed in a non-woven bag. Figure 4 shows a photograph of the radioprotective side shield attached to the radioprotective glasses (PANORAMA SHIELD HF-480S). The total weight of the shield was 15.4 g. The shield was attached to the temple of the radioprotective glasses. In addition, to help the shield conform to the shape of the face and maintain its shape, 0.4-mm thick steel wires were attached along the perimeter of the sheet. The height of the endoscopist phantom was varied between 150, 165, and 180 cm, and the phantom was rotated from 15–75° at 15° intervals. The DR[%] was calculated using Eq. (1).

## Results

### Number of sheet layers and eye lens dose

**Fig. 5:**
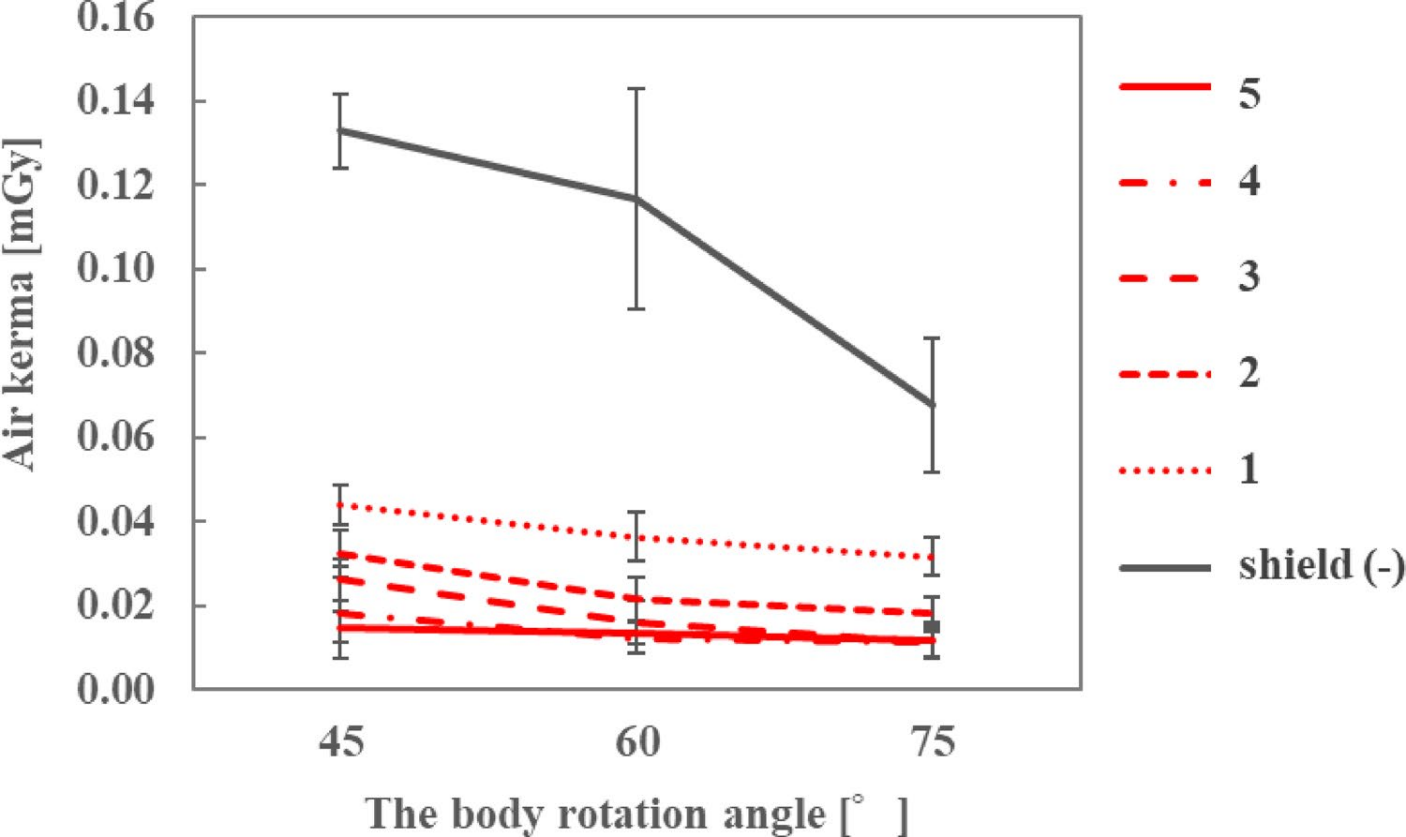
Air kerma for different sheet numbers when the height of the endoscopist phantom was 165 cm

The air kerma at the ocular surface of the endoscopist phantom and the DR[%] when the number of sheets was changed are shown in Fig. 5; Table 1, respectively. By stacking the sheets and increasing the lead equivalent, the air kerma decreased and the DR[%] was > 80% when three or more sheets were stacked.

**Table 1:** Dose reduction rate for different sheet numbers when the height of the endoscopist phantom was 165 cm

	Number of sheets				
	1	2	3	4	5
Body rotation angle [° ]	Dose reduction rate [%]				
45	67.0	75.8	80.3	86.3	88.9
60	68.9	81.5	86.4	89.6	88.7
75	53.4	73.4	83.2	83.3	82.9

### Length of the front side of the sheet and eye lens dose

**Fig. 6:**
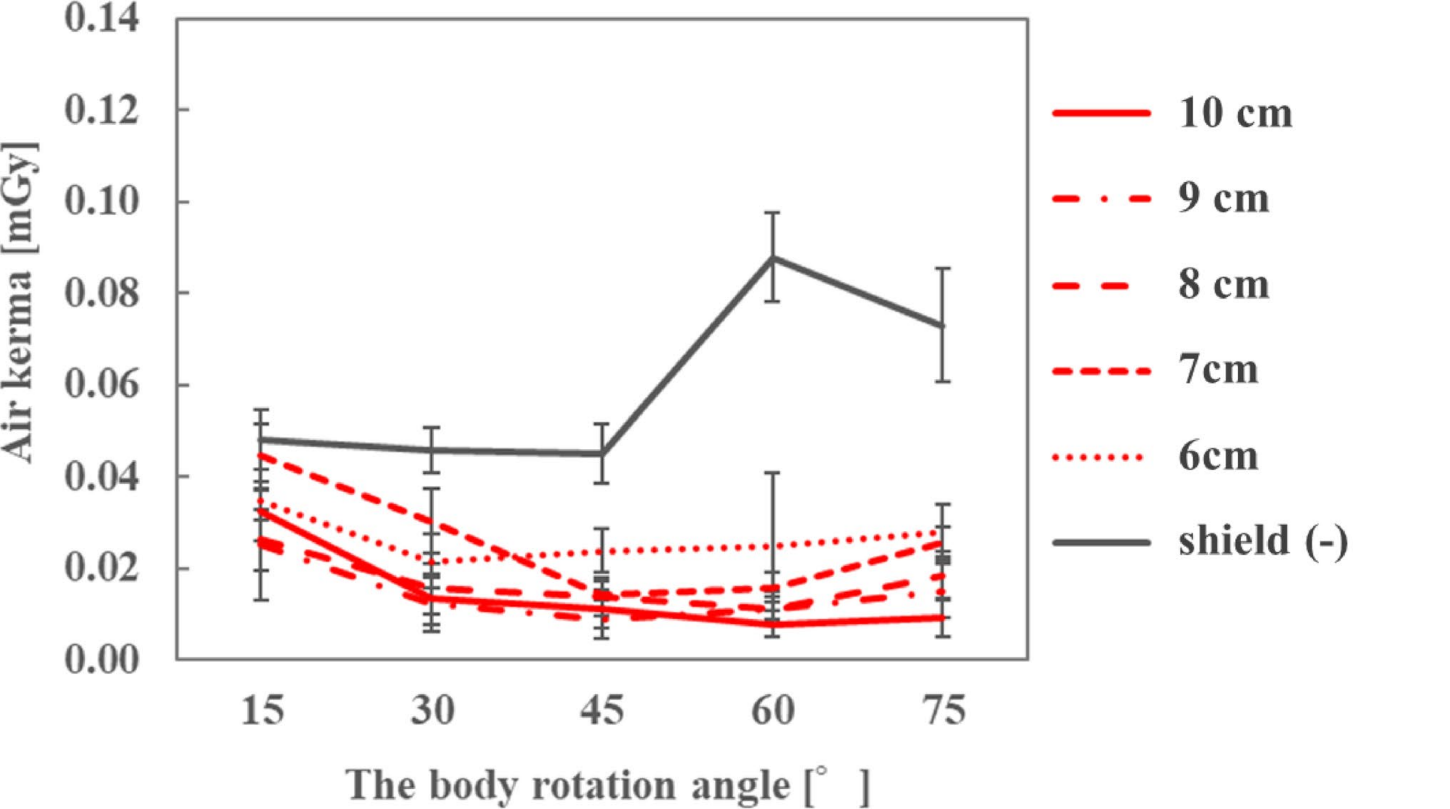
Air kerma for different front side lengths of the sheet when the height of the endoscopist phantom was 150 cm

**Fig. 7:**
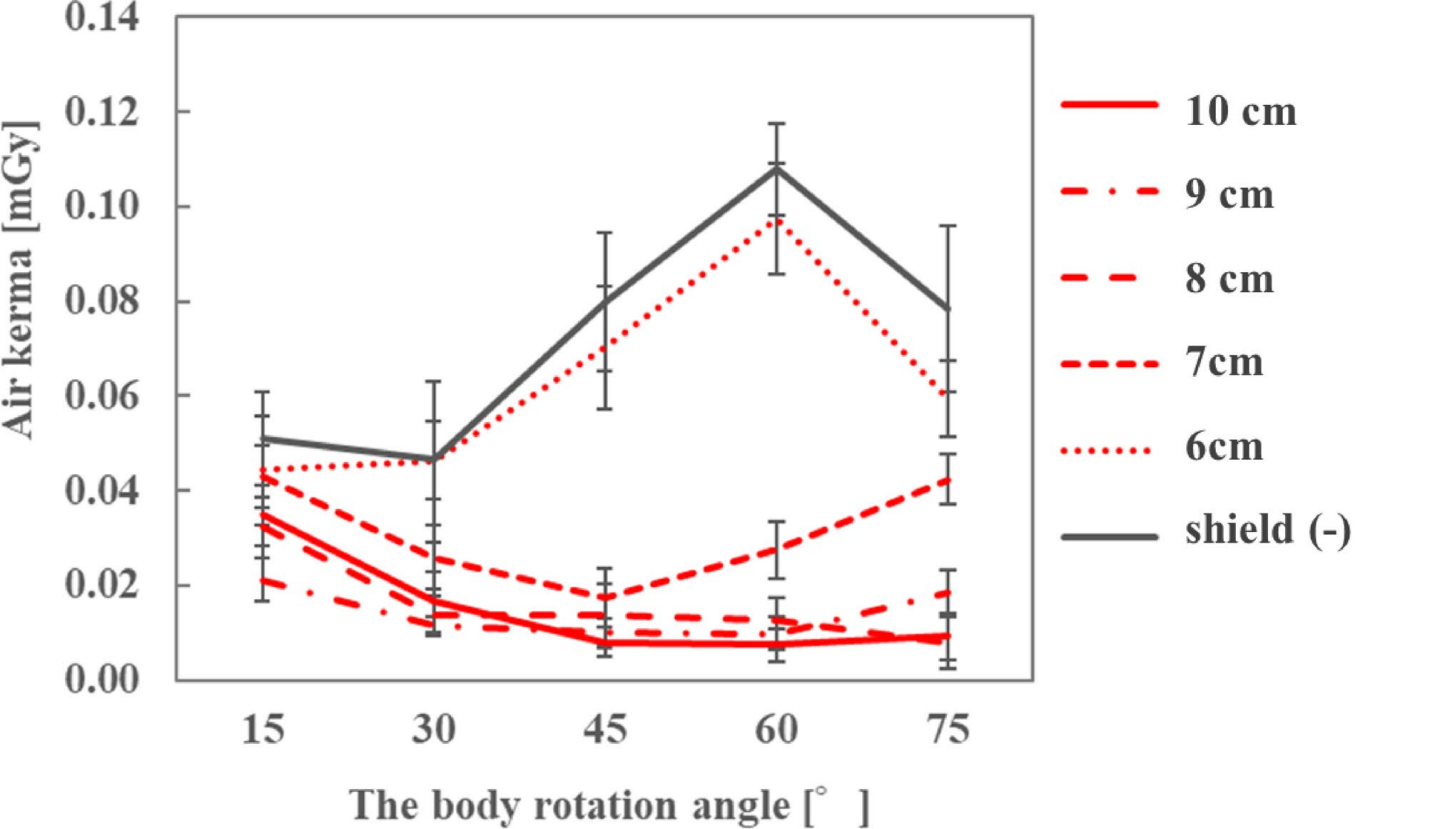
Air kerma for different front side lengths of the sheet when the height of the endoscopist phantom was 165 cm

**Fig. 8:**
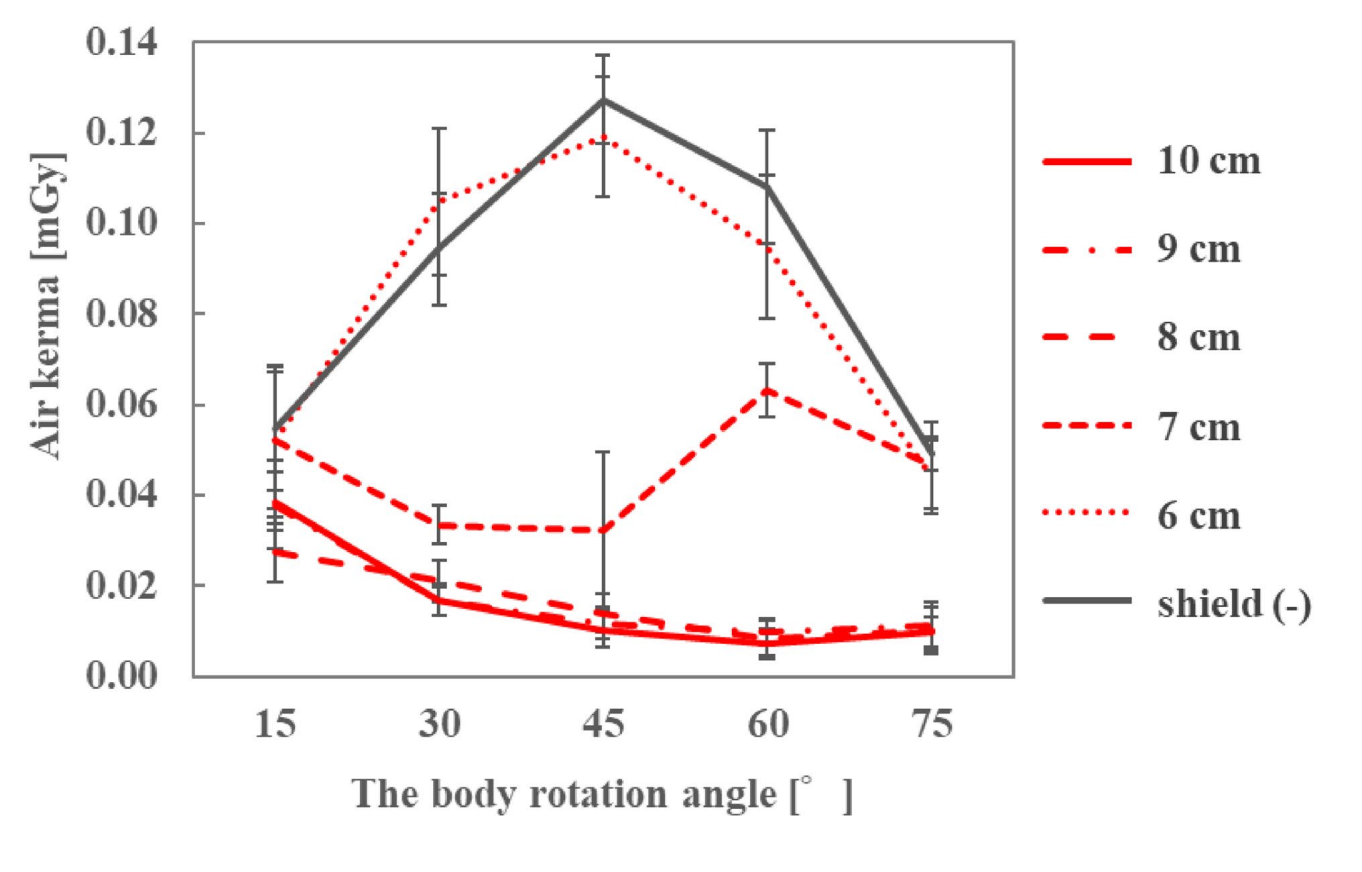
Air kerma for different front side lengths of the sheet when the height of the endoscopist phantom was 180 cm

The air kerma on the ocular surface of the endoscopist phantom and the DR[%] with different front-side lengths of the sheet are shown in Figs. 6, 7 and 8; Tables 2, 3 and 4, respectively, for each phantom height. At a height of 165 cm, the air kerma without the sheet at the 45° position was 39.8% lower than that in Section 3.1. At all heights of the endoscopist phantom, the air kerma when the sheets were used with radioprotective glasses was lower than when only radioprotective glasses were used, and the DR[%] increased as the front-side length of the sheet increased. In particular, when the front-side length of the sheet was ≥ 8 cm, the air kerma on the ocular surface was < 0.02 mGy.

**Table 2:** Dose reduction rate for different front side lengths of the sheet when the height of the endoscopist phantom was 150 cm

	Front side length of the sheet [cm]				
	6	7	8	9	10
Body rotation angle [° ]	Dose reduction rate [%]				
15	27.8	7.6	45.5	47.9	32.7
30	52.9	33.7	66.0	73.3	71.2
45	47.2	68.6	69.4	80.3	75.7
60	71.8	82.1	87.2	87.2	91.2
75	61.6	64.9	74.9	79.4	87.4

**Table 3:** Dose reduction rate for different front side lengths of the sheet when the height of the endoscopist phantom was 165 cm

	Front side length of the sheet [cm]				
	6	7	8	9	10
Body rotation angle [° ]	Dose reduction rate [%]				
15	13.5	16.0	37.1	58.6	31.8
30	0.9	44.4	70.8	75.4	64.5
45	12.1	78.2	82.7	87.6	89.9
60	9.5	74.4	88.3	90.9	93.1
75	24.2	46.0	90.1	76.6	88.3

**Table 4:** Dose reduction rate for different front side lengths of the sheet when the height of the endoscopist phantom was 180 cm

	Front side length of the sheet [cm]				
	6	7	8	9	10
Body rotation angle [° ]	Dose reduction rate [%]				
15	4.7	4.4	49.9	30.6	29.2
30	-11.1	64.5	77.7	82.4	82.0
45	6.4	74.6	89.1	90.8	91.9
60	12.5	41.6	92.2	90.9	93.4
75	10.4	5.1	79.2	77.5	80.1

### Length of the rear side of the sheet and eye lens dose

**Fig. 9:**
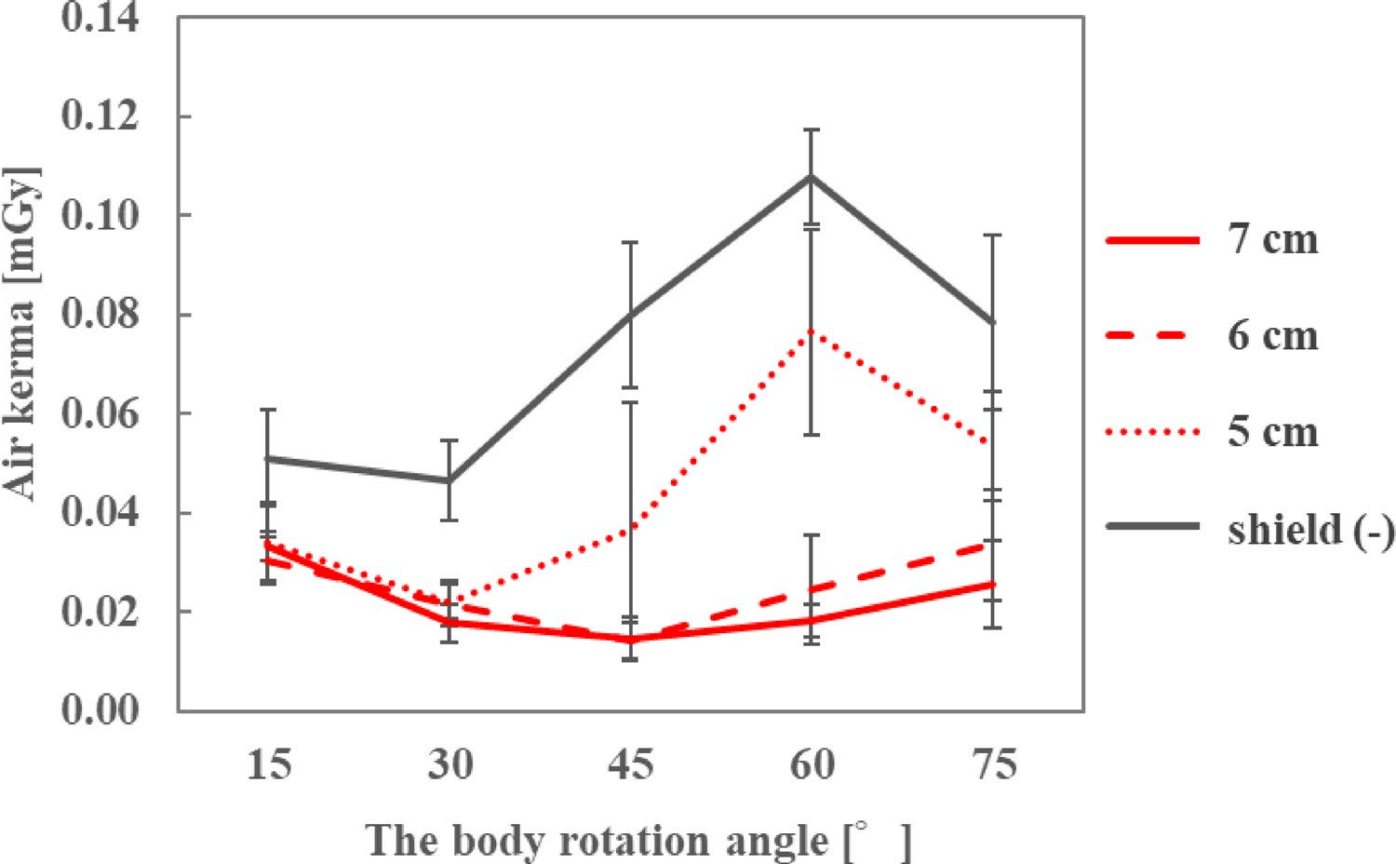
Air kerma for different rear side lengths of the sheet when the height of the endoscopist phantom was 165 cm

**Fig. 10:**
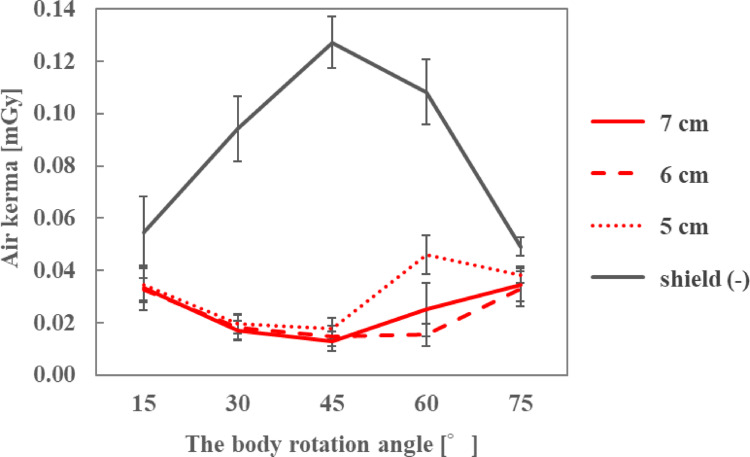
Air kerma for different rear side lengths of the sheet when the height of the endoscopist phantom was 180 cm

The air kerma on the ocular surface of the endoscopist phantom and the DR[%] with different rear-side lengths of the sheet are shown in Figs. 9 and 10; Tables 5 and 6, respectively, for each phantom height. At both heights of the endoscopist phantom, the air kerma when the sheets were used with radioprotective glasses was lower than that when only radioprotective glasses were used. The DR[%] increased as the rear-side length of the sheet increased. In particular, when the phantom height was 165 cm and the rear-side length of the sheet was 5 cm, the air kerma on the ocular surface increased as the phantom rotation angle increased. However, when the rear-side length was increased to 6 and 7 cm, the air kerma on the ocular surface decreased, and the DR[%] was approximately ≥ 60%.

**Table 5:** Dose reduction rate for different Rear side lengths of the sheet when the height of the endoscopist phantom was 165 cm

	Rear side length of the sheet [cm]		
	5	6	7
Body rotation angle [° ]	Dose reduction rate [%]		
15	33.1	40.5	34.4
30	52.4	53.2	63.0
45	54.4	82.2	81.7
60	29.0	77.1	82.9
75	31.7	57.2	67.2

**Table 6:** Dose reduction rate for different Rear side lengths of the sheet when the height of the endoscopist phantom was 180 cm

	Rear side length of the sheet [cm]		
	5	6	7
Body rotation angle [° ]	Dose reduction rate [%]		
15	36.6	39.9	38.5
30	79.3	80.8	81.7
45	86.1	88.3	89.8
60	57.5	85.7	76.8
75	22.1	32.7	30.1

### Dose reduction effect of radioprotective side shield

**Fig. 11:**
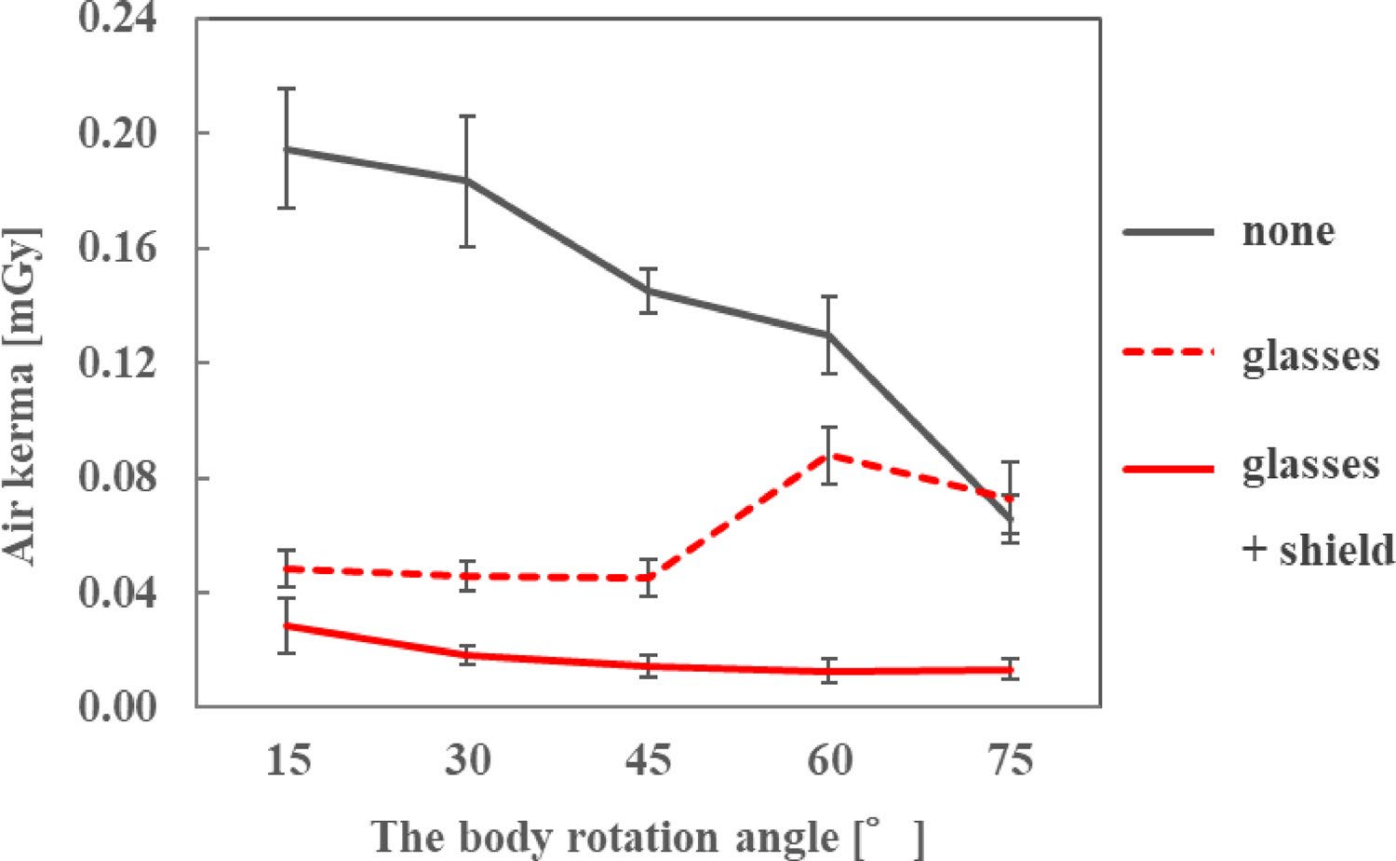
Air kerma with and without a radioprotective side shield when the height of the endoscopist phantom was 150 cm

**Fig. 12:**
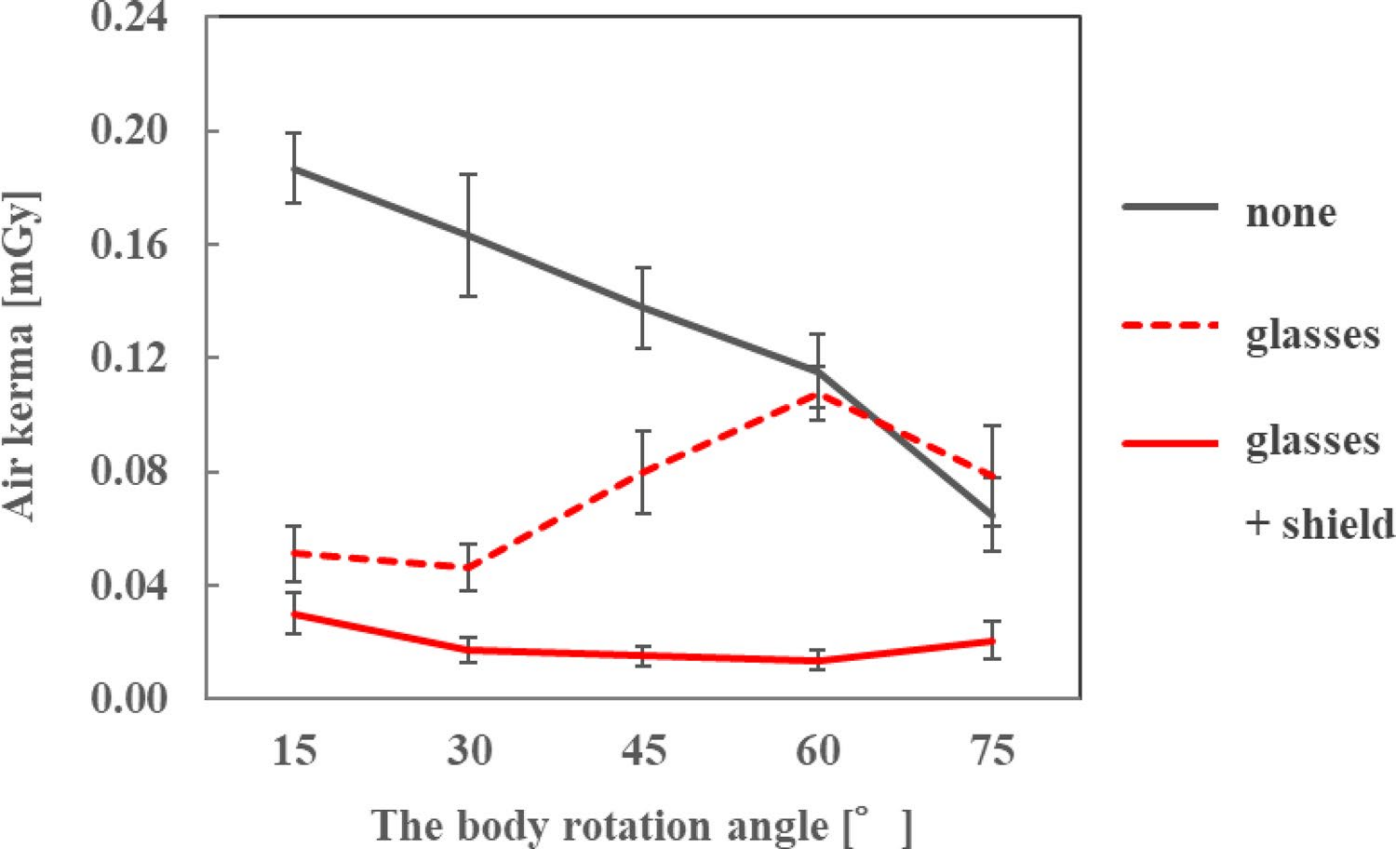
Air kerma with and without a radioprotective side shield when the height of the endoscopist phantom was 165 cm

**Fig. 13:**
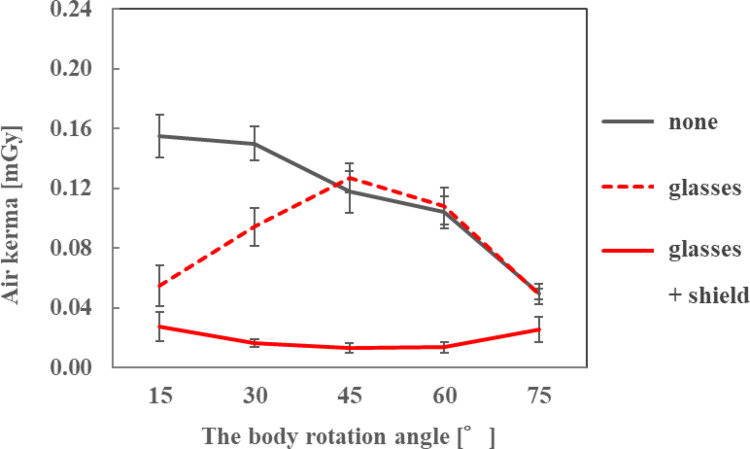
Air kerma with and without a radioprotective side shield when the height of the endoscopist phantom was 180 cm

The air kerma on the ocular surface of the endoscopist phantom and the DR[%] when the radioprotective side shield was attached to the radioprotective glasses are shown in Figs. 11, 12 and 13; Table 7, respectively, for each phantom height. At all heights of the endoscopist phantom, the air kerma on the ocular surface when attaching the radioprotective side shield to the radioprotective glasses was smaller than that when wearing nothing or only radioprotective glasses. The addition of the side shield reduced the air kerma to approximately 0.02 mGy, achieving dose reduction rates of approximately 40–90% compared to radioprotective glasses alone. When only radioprotective glasses were worn, the air kerma tended to increase as the phantom rotation angle increased. However, when the radioprotective side shield was attached to the radioprotective glasses, the air kerma decreased to approximately 0.02 mGy regardless of the phantom rotation angle.

**Table 7:** Dose reduction rate of the radioprotective side shield

	Height of the endoscopist phantom [cm]		
	150	165	180
Body rotation angle [° ]	Dose reduction rate [%]		
15	40.8	41.0	48.9
30	60.2	62.7	82.6
45	68.2	81.0	89.6
60	85.5	87.2	87.3
75	81.7	73.6	48.2

## Discussion

During fluoroscopy-guided endoscopic procedures such as ERCP, the radiation dose to the endoscopist’s eye lens may be high even if the endoscopist is wearing radioprotective glasses, due to a gap between the face and the radioprotective glasses, through which scattered X-rays can enter. Therefore, in this study, we developed a radioprotective side shield that can be attached to radioprotective glasses and evaluated its dose reduction effect at the ocular surface.

When olefin resin sheets containing Bi_2_O_3_ were attached to the temple of the radioprotective glasses, the air kerma on the ocular surface of the endoscopist phantom was reduced compared with when only the radioprotective glasses were worn. Furthermore, the more the layers of sheets used and the greater the lead equivalent of the sheets, the lower was the air kerma on the ocular surface and the higher was the DR[%], compared with using radioprotective glasses alone. In particular, when three or more sheets were stacked, the air kerma was < 0.02 mGy, achieving a DR[%] of > 80% regardless of the phantom rotation angle. A study by Hu et al. [24] revealed that an increase in the lead equivalent of radioprotective glasses above 0.35 mmPb did not result in a significant increase in the DR[%]. In this study, the DR[%] did not increase significantly, even when more than three sheets were stacked, suggesting that lighter sheets are more comfortable to use for long periods; therefore, stacking more than three sheets is not required. However, while this study was based on actual measurements, the referenced literature relied on simulation. In that reference, only the front lenses of the glasses were modeled. Due to differences in geometry, the incident angle of scattered X-rays and the extent of the shielded area may differ, which likely explains the differences in lead thickness.

As the length of the front side of the sheet increased, the air kerma on the ocular surface decreased, resulting in a higher DR[%] compared with using radioprotective glasses alone. This is because the longer the sheet, the smaller is the gap below the eye lens, reducing the amount of patient phantom-generated scattered X-rays entering through the gap at the bottom. For the endoscopist phantom with a height of 150 cm, the air kerma at the ocular surface was < 0.03 mGy even with a minimum sheet length of 6 cm. However, for phantom heights of 165 and 180 cm, the air kerma on the ocular surface when using the 6-cm sheet was higher than when using sheets longer than 6 cm. In particular, at a phantom height of 180 cm, the air kerma with the 6-cm sheet was almost equivalent to that without the sheet. In a previous study [25], the eye lens dose was higher when the height of the medical staff was higher because more scattered X-rays reached the eye lens of the medical staff from the lower front gap during CT. Similarly, in this study, the higher the endoscopist phantom, the more was the amount of scattered X-rays that entered through the gap at the bottom of the radioprotective glasses. By increasing the front-side length of the sheet to ≥ 8 cm, the air kerma on the ocular surface was < 0.02 mGy regardless of the height of the endoscopist phantom, achieving a DR[%] of > 80% under most conditions. At a height of 165 cm, the air kerma without the sheet at the 45° position was 39.8% lower than that in Section 3.1. This difference is attributed to the radioprotective glasses: the glasses used in the experiment described in Section 3.2. had larger side lenses than those used in Section 3.1, thereby reducing the scattered X-rays reaching the eyes and resulting in a lower dose.

As the rear-side length of the sheet increased, the air kerma on the ocular surface position decreased, resulting in a higher DR[%] compared with using radioprotective glasses alone. Mao et al. [26] showed that as the endoscopist’s body rotation angle increased, the lateral gap between the face and radioprotective glasses was more oriented toward the source of the scattered X-rays, allowing more of these to reach the eye lens through this gap, whereby the radioprotective glasses provided less protection for the eye lens. In contrast, in this study, the longer the rear-side length of the sheet, the more effectively it was possible to prevent scattered X-rays from reaching the eye lens from the posterior inferior diagonal side. This is because the air kerma on the ocular surface decreases regardless of the phantom rotation angle. However, when the endoscopist phantom height was 180 cm, the air kerma did not decrease considerably regardless of the presence or length of the shield. This is because the higher the endoscopist’s phantom, the smaller the effect of the rear length of the sheet on the air kerma, with scattered X-rays mainly entering from the bottom rather than from the posterior inferior diagonal. By increasing the length of the rear side of the sheet to ≥ 6 cm, the air kerma was < 0.04 mGy regardless of the height of the endoscopist phantom, and a DR[%] of > 70% was achieved under most conditions.

Based on the verification results, three olefin resin sheets containing Bi_2_O_3_, each 8-cm long at the front and 6-cm long at the rear, enclosed in a non-woven fabric cover, were used as radioprotective side shields. In an evaluation using a phantom representing an endoscopist performing ERCP, the radioprotective side shield reduced the air kerma to approximately 0.02 mGy, regardless of the height or orientation angle of the endoscopist phantom. Furthermore, the addition of the side shield resulted in dose reduction rates of approximately 40–90% compared to radioprotective glasses alone. This is because the radioprotective side shield can prevent scattered X-rays coming from below or diagonally behind the endoscopist phantom from entering through the gap between the endoscopist phantom’s face and the radioprotective glasses. Furthermore, freely deformable steel wires of approximately 0.4 mm thickness were attached to the outer periphery of the nonwoven fabric cover, allowing the sheets to fit closely to the face of the endoscopist phantom and reducing gaps, thereby reducing the incidence of scattered X-rays. Martin [27] showed that the majority of radiation exposure to the eye lens comes from radiation scattered from the irradiated tissues surrounding the eye. However, the radioprotective side shield evaluated in this study protected the temple and cheek on the covered side, thereby reducing the amount of incoming scattered X-rays. For the endoscopist phantom with a height of 165 cm, the average air kerma on the ocular surface was 0.134, 0.073, and 0.019 mGy when nothing was worn, when only radioprotective glasses were worn, and when both radioprotective glasses and side shield were worn, respectively. These values can be converted to *H*_p_(3) of 0.171, 0.093, and 0.025 mSv, respectively, using an *h*_p*K*_(3) of 1.28 Sv/Gy calculated from a cylindrical International Commission on Radiation Units and Measurements tissue phantom for a narrow spectrum series of N-40 and an incident angle of 0° [28]. According to a study by Takenaka et al. [29], the average ERCP fluoroscopy time was 14.88 ± 10.79 min. Assuming that the fluoroscopy time per procedure was 15 min, the cumulative *H*_p_(3) values were calculated to be 0.256, 0.140, and 0.037 mSv, respectively. The estimated numbers of examinations performed per week within 20 mSv/yr were 1, 2, and 10, respectively. Radioprotective side shields can significantly increase the number of ERCP procedures performed below the equivalent dose limit. In addition, it is lightweight (only 15.4 g) and highly versatile because it has been confirmed to be attachable to radioprotective glasses with various lens sizes, including over-glasses and goggle types. Therefore, it can be attached to any type of radioprotective glasses unless the glasses have a uniquely shaped frame. Moreover, the side shield provided sufficient protective effects for two types of radioprotective glasses: Dr. View X-RAY type OG, which has a high lead equivalent (0.85 mmPb) but small side lenses and no lower lens, and PANORAMA SHIELD HF-480S, which has a low lead equivalent (0.07 mmPb) but large side and lower lenses. Therefore, similar protective effects can be expected when the side shield is used with other radioprotective glasses that have comparable lens shapes and lead equivalents. It thus represents a novel approach to reduce the endoscopist’s eye lens dose during ERCP.

This study had certain limitations. First, it was a phantom study. Furthermore, it is worth noting that the endoscopist’s body orientation, standing position, and head direction can change at any time during ERCP; however, these effects were not considered in this study. Furthermore, the effects of the radioprotective side shield on endoscopists, such as comfort, visual field obstruction, ease of sterilization/cleaning, and likelihood of compliance, were not verified. Second, this study used a specific fluoroscopy X-ray system and phantoms, and evaluated only one type of fluoroscopy condition, which may have affected the results. In this study, the side shield was evaluated using only two types of radioprotective glasses with different lens shapes and lead equivalents. Although sufficient protective effects were demonstrated for both types, further studies are required to validate its applicability to other models of radioprotective glasses. Finally, the *H*_p_(3) conversion factor *h*_p*K*_(3) is generally applied by multiplying with the air kerma without backscattering [28]. However, the readings of the small OSL dosimeters in the present study were affected by backscattered X-rays from the radioprotective glasses and phantom, which may have led to an overestimation of *H*_p_(3).

## Conclusion

In this study, a radioprotective side shield that can be attached to radioprotective glasses was developed and its dose reduction effect on eye lens in a phantom study simulating ERCP was evaluated. Air kerma on the ocular surface varied with the number and size of sheets. By increasing the front-side length of the sheets to 8 cm, their rear-side length to 6 cm, and the number of sheets to three, the air kerma on the ocular surface was considerably reduced. The use of a radioprotective side shield made of three layers of sheets with a lead equivalent of 0.14 ± 0.02 mmPb, each 8-cm long at the front and 6-cm long at the rear, enabled the air kerma on the ocular surface of the endoscopist phantom to be reduced to approximately 0.02 mGy during 10-min fluoroscopy simulating ERCP, regardless of the phantom height or orientation. These results highlight the use of a radioprotective side shield as a novel approach to reduce the exposure of the eye lens of endoscopists during ERCP.

## Data Availability

Data cannot be made publicly available upon publication because no suitable repository exists to host them. Data supporting the findings of this study are available upon reasonable request from the authors.
